# Altered Gut Microbial Fermentation and Colonization with *Methanobrevibacter smithii* in Renal Transplant Recipients

**DOI:** 10.3390/jcm9020518

**Published:** 2020-02-14

**Authors:** Tim J. Knobbe, Rianne M. Douwes, Daan Kremer, J. Casper Swarte, Michele F. Eisenga, António W. Gomes-Neto, Marco van Londen, Frans T. M. Peters, Hans Blokzijl, Ilja M. Nolte, Wouter H. Hendriks, Hermie J. M. Harmsen, Stephan J. L. Bakker

**Affiliations:** 1 Department of Internal Medicine, Division of Nephrology, University Medical Center Groningen, University of Groningen, 9700 RB Groningen, The Netherlands; r.m.douwes@umcg.nl (R.M.D.); d.kremer@umcg.nl (D.K.); j.c.swarte@umcg.nl (J.C.S.); m.f.eisenga@umcg.nl (M.F.E.); a.w.gomes.neto@umcg.nl (A.W.G.-N.); m.van.londen@umcg.nl (M.v.L.); s.j.l.bakker@umcg.nl (S.J.L.B.); 2 Department of Gastroenterology and Hepatology, University Medical Center Groningen, University of Groningen, 9700 RB Groningen, The Netherlands; f.t.m.peters@umcg.nl (F.T.M.P.); h.blokzijl@umcg.nl (H.B.); 3 Department of Epidemiology, University Medical Center Groningen, University of Groningen, 9700 RB Groningen, The Netherlands; i.m.nolte@umcg.nl; 4 Department of Animal Sciences, Wageningen University & Research centre, 6708 PB Wageningen, The Netherlands; wouter.hendriks@wur.nl; 5 Department of Medical Microbiology, University Medical Center Groningen, University of Groningen, 9700RB Groningen, The Netherlands; h.j.m.harmsen@umcg.nl

**Keywords:** posttransplant diarrhea, methanogenesis, *Methanosphaera stadtmanae*, mucins, sulfate-reducing bacteria

## Abstract

Renal transplant recipients (RTRs) often suffer from posttransplant diarrhea. The observed dysbiosis in RTR may influence the fermentation processes in the gut. In this study, we aimed to investigate whether fermentation differs between RTRs and healthy controls (HCs), by measuring breath H_2_ and CH_4_ concentrations. Additionally, we determined the fecal presence of the methanogen *Methanobrevibacter smithii* (*M. smithii*), which plays a main role in the process of methanogenesis. Data from the TransplantLines Biobank and Cohort Study (NCT03272841) was used. A total of 142 RTRs and 77 HCs were included. Breath H_2_ concentrations in RTRs were not significantly different from HCs. Breath CH_4_ concentrations in RTRs were significantly lower compared with HCs (median [interquartile range (IQR)] 7.5 [3.9–10.6] ppm vs. 16.0 [8.0–45.5] ppm, *p* < 0.001). *M. smithii* was less frequently present in the feces of RTRs compared to HCs (28.6% vs. 86.4% resp., *p* < 0.001). Our findings regarding the altered methanogenesis in the gut of RTRs show similarities with previous results in inflammatory bowel disease patients. These findings provide novel insight into the alterations of fermentation after renal transplantation, which may contribute to understanding the occurrence of posttransplant diarrhea.

## 1. Introduction

Renal transplantation is the preferred treatment for patients with end-stage renal disease [1,2,3]. Part of its success has been made possible by improved therapeutic options, such as ameliorations in surgical techniques and perioperative care [4]. Despite the success of transplantation, the burden of morbidity in renal transplant recipients (RTRs) remains high [5].

Patients often experience gastrointestinal complaints such as diarrhea, which is associated with premature kidney allograft failure and mortality, and which affects quality of life [5,6]. This posttransplant diarrhea is believed to be non-infectious and induced by the use of medication [5,7]. Recently, a study in RTRs showed that dysbiosis in the gut might cause or contribute to this posttransplant diarrhea [7]. Lee et al. demonstrated in this study that the gut microbiota diversity of RTRs with diarrhea was significantly lower than in RTRs without diarrhea. In addition, RTRs with diarrhea had a lower diversity of commensal bacterial taxa in the gut, creating a dysfunctional metabolic state. These commensal bacterial taxa are important for the degradation of complex molecules such as complex carbohydrates. During this degradation, among many other molecules, short-chain fatty acids are produced, which contributes to overall gut health [8,9]. It has been proposed that posttransplant diarrhea might be the consequence of a diminished ability to digest complex sugars [7]. A proportion of complex polymers such as fibers escape digestion and absorption in the small bowel. These complex polymers are then fermented to short-chain fatty acids (acetate, butyrate and propionate) and gases (hydrogen (H_2_) and carbon dioxide (CO_2_)) [10].

In order to maintain fermentation, it is essential that H_2_ concentration is reduced by H_2_-consuming microorganisms [11]. H_2_ can be used as an electron donor in sulfate respiration, methanogenesis or acetogenesis to produce hydrogen sulfide (H_2_S), methane (CH_4_) and acetate, respectively [12]. Production of H_2_S is most favorable, followed by the production of CH_4_ and acetate, respectively. However, for the production of H_2_S, the presence of sulfate is necessary [13]. The production of CH_4_ is performed by archaea. *Methanobrevibacter smithii* (*M. smithii*) and *Methanosphaera stadtmanae (M. stadtmanae)* are the two methanogens usually detected in the human gut. *M. smithii* is the predominant methanogen in the human colon [14]. Next to H_2,_, formate can be used for the methanogenesis as well [15]. The produced CH_4_ and the remaining H_2_ are excreted in breath and flatus. Therefore, both gases can be measured in exhaled breath [13]. Measuring breath CH_4_ concentrations is a simple way to investigate the metabolism of intestinal methanogens, since no significant catabolism elsewhere in the human body has been observed [12]. The presence of *M. smithii* can be measured in the feces, as has previously been performed in studies investigating patients suffering from inflammatory bowel disease (IBD) [12,16].

The dysbiosis in RTRs may influence the fermentation in the gut and the processes following fermentation, possibly leading to or contributing to posttransplant diarrhea. To gain more insight into pathogenesis of this diarrhea, we aimed to investigate the fermentation and methanogenesis in the gut in RTRs. Firstly, we aimed to investigate whether breath H_2_ and CH_4_ concentrations differ between RTRs and HCs. Secondly, we aimed to investigate whether the presence of *M. smithii* in feces differs between RTRs and HCs, and finally we aimed to identify the determinants of CH_4_ production.

## 2. Methods

### 2.1. Study Population

For this study we used data from the TransplantLines Biobank and Cohort Study (ClinicalTrials.gov identifier: NCT03272841). A detailed description of the study design, inclusion and exclusion criteria has been described previously [17]. In addition to the standard protocol, we measured breath H_2_ and CH_4_ concentrations and analyzed the presence of *M. smithii* in feces for the current study. (Potential) living organ donors were used as a healthy control group for comparison. Our inclusion period was between February and December 2017. The study protocol has been approved by the Institutional Review Board (METc 2014/077) (METc UMCG), adheres to the UMCG Biobank Regulation, and is in accordance with the WMA Declaration of Helsinki and the Declaration of Istanbul [17].

### 2.2. Patient Comorbidities

Diabetes mellitus was defined according to the guidelines of the American Diabetes Association [18]. The estimated glomerular filtration rate (eGFR) was calculated using the serum creatinine-based chronic kidney disease epidemiology collaboration (CKD-EPI) formula. Data regarding the history of allograft rejection and primary renal disease before transplantation were retrieved from patients’ medical files.

### 2.3. Breath H_2_ and CH_4_ Concentration Measurement

For H_2_ and CH_4_ measurements, breath samples were collected using a 50 cc syringe with an opening of 6 mm in diameter at approximately 40 cc with a 3-way-stopcock. Subjects were instructed to inhale normally and exhale fully in the syringe, with the plunger set at 50 cc and the 3-way stopcock open. After full expiration, the opening was immediately closed by the subject’s finger, the plunger was set to 30 cc and the 3-way stopcock was closed. This resulted in breath samples that were not diluted by environmental air. Two breath samples were taken subsequently per study subject. Breath samples were analyzed within 12 h after sample collection. H_2_, CH_4_ and CO_2_ measurements were performed using a solid-state gas-chromatography device (Breathtracker SC, QuinTron Instrument Company, Inc., Milwaukee, WI, USA). The device separates the components by the basic principle of gas chromatography, using room air as the carrier gas, which is pumped through the system by an internal circulating pump. H_2_ and CH_4_ are separated from all other reducing gases and from each other, and are carried past a solid-state sensor [19]. The sensors are reported to be affected only by reducing gases, so it is unaffected by other gases in the sample; it can also employ a CO_2_ correction factor [19]. The analytical sensitivity is 1 ppm for H_2_ and CH_4_ and 0.1% for CO_2_. The Breathtracker has a linear analytical range of 2–150 ppm for H_2_, 2–75 ppm for CH_4_ and 1000–70,000 ppm for CO_2_. To ensure reliable breath measurements, study subjects were not allowed to smoke for at least one hour before the sample collection [20].

### 2.4. M. Smithii Measurement in Feces

Fecal samples were collected the day prior to the TransplantLines visit, using a FecesCatcher (TAG Hemi VOF, Zeijen, The Netherlands) and were immediately frozen after collection. The feces samples were transported in cold storage to the TransplantLines visit, and immediately stored at −80 ℃ (−112 ˚F) [17]. After thawing, DNA was extracted with the RBB and Qiagen method, as performed by Yu et al. with modifications described by de Goffau et al. [21,22]. To measure the quantity of *M. smithii*, real-time quantitative reverse transcription polymerase chain reaction (RT-PCR) (7500 real time PCR system, applied Biosystems, Thermo Fisher Scientific, Waltham, USA) was performed. Primers were taken as described by Johnston et al., and differentiation between *M. smithii* and other organisms in the sample was assessed using *nifH* genes [23]. The number of *nifH* genes are equal to the number of *M. smithii*, since only one gene of *nifH* is present in each *M. smithii* [24]. Analyses were performed using the Taqman machine and processed using SDSShell (Applied Biosystems, Thermo Fisher Scientific, Waltham, USA). The quantifiable presence of *M. smithii* was determined using a cycle threshold value. Values < 40 cycles were regarded as positive, and values ≥40 were regarded as negative. For analyses, CT-values ≥40 were regarded as negative and concentrations of *M. smithii* in these patients were regarded as 0 *M. smithii*/gram feces. A detailed method description is attached in Supplementary File 1.

### 2.5. Statistical Analyses

Statistical analyses were performed using the Statistical Package for the Social Sciences (SPSS) version 23.0 (IBM corp.; Armonk, NY, USA). In all analyses, *p* < 0.05 was regarded as statistically significant. Categorical variables are presented as *n* (%), normally distributed variables as mean ± standard deviation (SD) and non-normally distributed variables as median [interquartile range]. Normality was assessed using Q–Q plots. Differences between groups with normally distributed variables were assessed using independent T-tests. Non-normally distributed data were compared using the Mann–Whitney U test. Comparison of categorical variables was performed using a chi-square test for groups with *n* ≥ 5 and a Fisher’s exact test for groups with *n* < 5. For all other tests and visualizations, the mean of the duplicate measurements of the breath H_2_ and CH_4_ concentration in breath was used. To correct for environmental CH_4_, 2 ppm was subtracted from each breath CH_4_-measurement [25,26]. Possible determinants of breath CH_4_ were identified using univariable linear regression. All variables with a *p*-value <0.2 were included in a multivariable linear regression model run backward to identify the determinants of breath CH_4_ production. Because H_2_ is used by *M. smithii* for the conversion to CH_4_, an interaction term of H_2_ and *M. smithii* was added in the analysis. Log_10_ transformations were performed if necessary to reach conditions in all performed analyses.

## 3. Results

**Figure 1:**
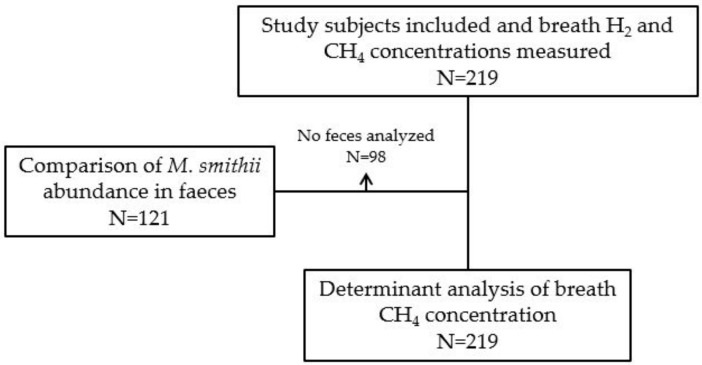
Consort flow diagram. Abbreviations: CH_4_, methane; H_2_, hydrogen; *M. smithii*, *Methanobrevibacter smithii.*

We included 219 study subjects, of whom 142 (64.8%) were RTRs and 77 (35.2%) were HCs. Among RTRs, 91 (64.1%) were male, and the mean age was 56.3 ± 13.7 years. Among HCs, 39 (50.6%) were male, and the mean age was 56.4 ± 10.6 years. Baseline characteristics are shown in Table 1. A Consort Flow diagram is presented to provide an overview of subgroups that were used in different analyses (Figure 1). Breath H_2_ concentrations of the RTRs were not significantly different compared with HCs (Table 1). The RTRs had, however, lower breath CH_4_ concentrations compared to the HCs (7.5 [3.9–10.6] ppm vs. 16.0 [8.0–45.5] ppm, *p* < 0.001). Data distributions of breath H_2_ and CH_4_ concentrations are shown in Supplementary File 2. Raw data are shown in the Supplementary data.

### 3.1. M. Smithii in Feces

The feces of 98 study subjects was not available for analysis. *M. smithii* abundance was analyzed in the feces samples of 77 RTRs and 44 HCs (i.e., 121 of 219 study subjects, see Figure 1). Among the RTRs, 22 (28.6%) had quantifiable concentrations of *M. smithii* in their stool samples. Among HCs, 38 (86.4%) had quantifiable concentrations of *M. smithii* in their feces samples. The median abundance of *M. smithii* in the feces of those study subjects was 5.9 × 10^7^ [1.2 × 10^6^–8.9 × 10^8^] per gram feces. A quantifiable concentration of *M. smithii* was significantly less frequently observed in RTRs compared to HCs ((22 (28.6%) vs. 38 (86.4%) resp.; *p* < 0.001) (Table 1). In addition, the abundance of *M. smithii* was positively correlated with breath CH_4_ concentrations (*r* = 0.69, *p* < 0.001).

### 3.2. Determinants of Breath CH_4_

**Figure 2:**
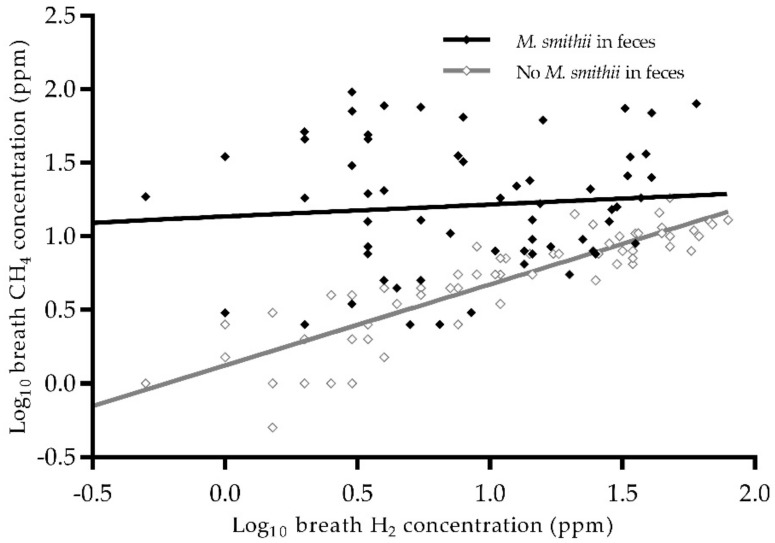
Scatterplot of log_10_ breath H_2_ and CH_4_ concentration by presence of *M. smithii* in feces. There is a difference between the relation between H_2_ and CH_4_ in subjects with and without *M. smithii*. Abbreviations: CH_4_, methane; H_2_, hydrogen; *M. smithii*, *Methanobrevibacter smithii*. *N* = 121. Pearson correlation in the absence of *M. smithii, r* = 0.88, *p* < 0.001. Pearson correlation in the presence of *M. smithii, r* = 0.09, *p* = 0.5.

Determinants of breath CH_4_ were analyzed using linear regression analysis in all 219 study subjects, and these results are presented in Table 2. Breath H_2_ and the presence of a quantifiable abundance of *M. smithii* in feces were associated with higher breath CH_4_ concentrations (standardized beta (st. β) 0.57, *p* < 0.001 and st. β 0.94, *p* < 0.001 resp.). A negative interaction was found between both determinants on breath CH_4_ (st. β −0.51, *p* = 0.001), indicating that in the presence of *M. smithii* the magnitude of the correlation between H_2_ and CH_4_ in breath decreases from overt to virtually absent (*r* = 0.88, *p* < 0.001 vs. *r* = 0.09, *p* = 0.5 resp., Figure 2). In addition, the use of mycophenolate mofetil was associated with a lower breath CH_4_ concentration (st. β −0.18, *p* = 0.014). The described determinants explained 55.0% of the total variation in breath CH_4_ concentrations.

## 4. Discussion

We have shown that although no significant difference in breath H_2_ concentration was found between RTRs and HCs, breath CH_4_ concentrations were significantly lower in the RTRs compared with the HCs. In addition, we found a significantly lower presence of *M. smithii* in the feces of RTRs compared with HCs. Breath H_2_ and the presence of *M. smithii* in feces were associated with higher breath CH_4_ concentrations. Moreover, the association between breath H_2_ and CH_4_ concentrations disappeared in presence of *M. smithii* in feces. Finally, mycophenolate mofetil was associated with a lower breath CH_4_ concentration.

The reduced breath CH_4_ concentration in RTRs compared to HCs which we observed might be explained by the reduced presence of *M. smithii* in the feces of RTRs, since *M. smithii* is the most abundant methanogen in the human gut [12]. One reason for the lower prevalence of *M. smithii* in the feces of RTRs may be the result of an increased presence or activity of sulfate-reducing bacteria (SRB). It has been described that a high concentration of either methanogens or sulfate-reducing bacteria is present in the feces of healthy individuals. These two groups of microorganisms appear to be competing for H_2_, with the prevailing group becoming the predominant organism [27,28]. However, since no mechanism of direct competition between SRB, methanogens and acetogens has been observed, at this point it is impossible to predict any dominance of one of these hydrogenotrophs [29]. If the gut in RTRs is more colonized with SRB, or if these SRB are more active, more hydrogen sulfide (H_2_S) is produced. H_2_S is highly toxic to the colonocytes and impairs their metabolic function, especially the butyrate oxidation [30]. Butyrate has a known anti-inflammatory effect and several other health-promoting functions [31]. The presence of butyrate in the lumen and the oxidation by colonocytes are both involved in the regulation of water and sodium absorption from the colon [30]. SRB and the consequent disturbance of butyrate oxidation is believed to play a key role in the pathogenesis of IBD [12]. If SRB are indeed more present in RTRs, this might also be an explanatory factor for the occurrence of any of the gastrointestinal complaints of RTRs [16], especially since the butyrate concentration in RTRs seems to be lower due to the reduced prevalence of bacteria taxa that produce butyrate [7].

In addition, more colonization with SRB, and consequently more H_2_S, may diminish the positive effects of the butyrate in the gut of RTRs. However, the presence of SRB was not measured in this study. Although our results regarding the decreased presence of *M. smithii* do support this hypothesis, future studies will have to further test this hypothesis.

Another reason for the lower prevalence of *M. smithii* in feces might be a lower presence of mucins in the gut of RTRs. We observed no significant correlation between breath H_2_ and CH_4_ concentrations in the presence of *M. smithii*. Therefore, the produced CH_4_ by *M. smithii* may be derived from endogenous substrates such as mucins, formate or other unknown substrates [12,32]. Importantly, for mucins it has not yet been settled whether they contribute to methane production or rather inhibit it, or under which circumstances stimulation may shift towards inhibition [32,33]. Mucins cover the epithelium and form a protective layer in the gut, thereby providing a protective layer against pathogenic organisms [34]. Deficiencies of mucin in the intestinal barrier are associated with an abnormal mucosal inflammatory response, which is present in IBD [34]. The role of mucins in the fermentation processes in RTRs has, to our best knowledge, never been investigated.

We also observed a strong correlation between breath H_2_ and CH_4_ concentrations in the absence of *M. smithii* in feces (Figure 2). Possibly, other methanogens that flourish in the absence of *M. smithii* are more dependent upon H_2_ concentrations. One of these methanogens might be *M. stadtmanae*, an archaeon that is the second most common archaeon in the healthy gut after *M. smithii* [35]. It is known that the CH_4_ production by *M. stadtmanae* is highly dependent upon the presence of H_2_ and methanol [36,37]. An increased prevalence of *M. stadtmanae* has been observed previously in IBD patients in a study by Lecours et al. [38]. Interestingly, it has been reported that *M. stadtmanae* can induce an inflammatory cytokine response from monocyte-derived dendritic cells, which may contribute to pathological conditions in the gut [39]. In order to gain more insight into gut health in RTRs, the prevalence of *M. stadtmanae* needs to be further investigated [12,39,40].

In addition, our study shows that patients using mycophenolate mofetil exhale lower concentrations of CH_4_. Previous studies have shown that mycophenolate mofetil is associated with gastrointestinal injury and diarrhea, although any underlying mechanisms are incompletely understood [6]. Lower CH_4_ levels are also associated with diarrhea [12]. Future studies may investigate whether methanogenesis plays a role in the association between mycophenolate mofetil and diarrhea.

Our observations are in line with previous studies in IBD patients [12,41]. This is interesting, since RTRs and IBD patients have similarities: both groups suffer from intestinal dysbiosis, often have diarrhea and often need to take immunosuppressive medication [38,42,43,44]. Scanlan et al. observed a significantly lower presence of methanogen-positive feces samples in patients suffering from ulcerative colitis compared to healthy controls (24% versus 48%). In addition, a lower presence of methanogens in patients suffering from Crohn’s disease was observed (30% versus 48%), although this association was not statistically significant [41]. In another study by Ghavami et al., significantly higher amounts of *M. smithii* were found in the feces samples of HCs compared to IBD patients [16]. Our results suggest that the reduced colonization with *M. smithii*, and possibly the methanogenesis of IBD patients, might be comparable to RTRs.

It is known that CH_4_ reduces inflammation, oxidative stress and apoptosis in the human body [45]. Our findings show significantly lower breath CH_4_ concentrations in RTRs compared to HCs, while the protective properties of CH_4_ appear especially important in RTRs in the context of (prevention of) renal rejection, inflammation and high levels of oxidative stress [46]. Future studies may further investigate the associations of (breath) CH_4_ concentrations with patient outcomes, such as renal rejection. If the hypotheses regarding the protective properties of CH_4_ are confirmed, the relatively low CH_4_ levels in RTR may be a therapeutic target, since CH_4_ concentrations in the body can be increased iatrogenically by inhalation or injection [47,48].

No difference in breath H_2_ concentration was found in the current study. This is in line with other studies: the matter of hydrogenotrophics in the gut is highly complex, and is dependent upon many variables [49].

It is a limitation of our study that we did not measure mucin concentrations or potential colonization by SRB or *M. stadtmanae*. In addition, we did not measure H_2_ and CH_4_ concentrations in flatus, although it has been found that the concentration of both gases are higher in flatus than in breath when the concentrations are high [50]. No data regarding menopausal status was available in our study, although it is known that menopausal status does affect the gut microbiome [51]. Additionally, we did not measure breath H_2_ and CH_4_ concentrations and the abundance of *M. smithii* in the feces of RTRs before transplantation. Further limitations of our study are that it was performed in a single center, and that our RTRs were included at different time points after transplantation. Another limitation of this study is that for logistical reasons we were unable to analyze *M. smithii* in the feces of all our study subjects. In addition, although we measured the breath H_2_ concentrations in the morning, shortly after breakfast, it was in a non-fasting state. Finally, the current study uses cross-sectional data, and therefore no conclusions regarding causal relationships can be drawn.

## 5. Conclusions

To the best of our knowledge, this is the first study to investigate both breath and feces samples in RTRs. The study shows that breath CH_4_ concentration and the prevalence of *M. smithii* in feces are significantly lower in RTRs compared to HCs. Our findings regarding the altered methanogenesis in the gut of RTRs show significant similarities with previous results in IBD patients. We observed that in the absence of *M. smithii*, breath CH_4_ production is highly dependent on H_2_ concentration, while this is not the case in the presence of *M. smithii*. Apparently, methanogenesis differs significantly depending on presence of *M. smithii*. Finally, the use of mycophenolate mofetil was associated with methanogenesis. These findings provide novel insight into the alterations of fermentation after renal transplantation, which may contribute to the occurrence of posttransplant diarrhea. In addition, this study has raised important hypotheses regarding the potential role of SRB and *M. stadtmanae* in post-transplant diarrhea. Future studies are needed to investigate the role of SRB and *M. stadtmanae.* Additionally, future research may study whether altered methanogenesis is associated with clinical outcomes, such as posttransplant diarrhea.

## Figures and Tables

**Table 1:** Baseline characteristics.

Characteristics	Renal Transplant Recipients	Healthy Controls	*p*-Value
Number of subjects, *n* (%)	142 (64.8)	77 (35.2)	*n*/a
Fermentation parameters
Breath H_2_ concentration, ppm	11.3 [4.0–30.0]	10.5 [4.5–28.3]	0.9
Breath CH_4_ concentration, ppm	7.5 [3.9–10.6]	16.0 [8.0–45.5]	<0.001
Quantifiable abundance of *M. smithii* in feces, *n* (valid %)	22 (28.6)	38 (86.4)	<0.001
Abundance of *M. smithii* in feces samples, *M. smithii*/gram	0.0 [0.0–4.0 × 10^5^]	5.9 × 10^7^ [1.2 × 10^6^–8.9 × 10^8^]	<0.001
Demographics
Age, y	56.3 ± 13.7	56.4 ± 10.6	0.6
Number of males, *n* (%)	91 (64.1)	39 (50.6)	0.05
BMI, kg/m^2^	28.0 ± 5.2	26.4 ± 3.8	0.01
Time since transplantation, y	1.0 [0.5–8.0]	-	n/a
Lifestyle parameters
Current smokers, *n* (valid %)	23 (16.7)	14 (18.9)	0.4
Alcohol intake per day, units	0.0 [0.0–0.2]	0.25 [0.0-0.5]	0.003
Laboratory parameters
Hemoglobin, g/dL	13.8 ± 1.9	14.4 ± 1.3	0.008
Hematocrit, L/L	0.42 ± 0.06	0.43 ± 0.04	0.2
Leukocytes, 10^9^/L	7.4 ± 2.5	6.5 ± 1.9	<0.003
Platelets, 10^9^/L	250.6 ± 78.4	261.2 ± 56.6	0.3
C-reactive protein, mg/L	2.3 [1.1–5.0]	1.2 [0.8–4.0]	0.035
Albumin, g/L	44.2 ± 3.1	45.4 ± 2.5	0.003
Glucose, mmol/L	6.0 ± 1.7	5.5 ± 0.7	0.005
HbA1c, mmol/mol	42.3 ± 7.8	36.9 ± 3.5	<0.001
eGFR, ml/min/1.73 m^2^	49.8 ± 16.5	69.3 ± 18.7	<0.001
Creatinine, µmol/L	130 [103.0–156.8]	92.0 [81.0–106.0]	<0.001
Urea, mmol/L	9.4 ± 4.4	5.8 ± 1.6	<0.001
Medication use
Antibiotics, *n* (%)	37 (16.9)	0 (0.0)	<0.001
Immunosuppressants, *n* (%)
Prednisolone, *n* (%)	140 (98.6)	-	*n*/a
Mycophenolate mofetil, *n* (%)	112 (78.9)	-	*n*/a
Tacrolimus, *n* (%)	102 (71.8)	-	*n*/a
Cyclosporine, *n* (%)	14 (9.9)	-	*n*/a
Everolimus, *n* (%)	7 (4.9)	-	*n*/a
Azathioprine, *n* (%)	10 (7.0)	-	*n*/a
Statins, *n* (%)	70 (49.3)	8 (10.4)	<0.001
Proton pump inhibitors, *n* (%)	108 (76.1)	0 (0.0)	<0.001
Insulin, *n* (%)	11 (7.8)	0 (0.0)	0.009
Biguanides, *n* (%)	7 (4.9)	0 (0.0)	0.09
Macrogol, *n* (%)	8 (5.6)	1 (1.3)	0.200
Lactulose, *n* (%)	2 (1.4)	0 (0.0)	0.500
Loperamide, *n* (%)	1 (0.7)	0 (0.0)	1.000
Antidepressants, *n* (%)	16 (7.3)	4 (5.2)	0.4
Primary renal disease before transplantation
Unknown, *n* (%)	23 (16.2)	-	*n*/a
Inflammatory disease	55 (38.7)
Congenital and hereditary kidney disease, *n* (%)	41 (28.9)	-	*n*/a
Renal vascular disease, excluding vasculitis, *n* (%)	13 (9.2)	-	*n*/a
Diabetic nephropathy, *n* (%)	10 (7.0)	-	*n*/a
Others
Diabetes mellitus, *n* (%)	27 (19.0)	1 (1.3)	<0.001
History of allograft rejection, *n* (%)	14 (9.9)	-	*n*/a

Data are presented as mean ± standard deviation (SD), median with interquartile ranges (IQRs) or number with percentages (%). Abbreviations: BMI, body mass index; eGFR, estimated glomerular filtration rate; CH_4_, methane; H_2_, hydrogen; HbA1c, hemoglobin A1c; *M. smithii*, *Methanobrevibacter smithii.*

**Table 2:** Linear regression analysis of log_10_ breath CH_4_ concentration.

	Univariable Linear Regression Analysis		Multivariable Linear Regression Analysis *	
	St. β	*p*-Value	St. β	*p*-Value
A medical history of renal transplantation (yes vs. no)	−0.42	<0.001		
Fermentation parameters				
Log_10_ Breath H_2_, ppm	0.32	<0.001	0.54	<0.001
Quantifiable abundance of *M. smithii* in feces (yes vs. no)	0.55	<0.001	0.95	<0.001
Interaction between log_10_ breath H_2_ and *M. smithii* in feces	0.48	<0.001	−0.51	0.001
Demographics				
Age, y	0.07	0.3		
Gender (yes vs. no)	−0.02	0.7		
BMI, kg/m^2^	−0.18	0.012		
eGFR, mL/min/1.73 m^2^	0.25	<0.001		
Intoxications				
Smoking (yes vs. no)	−0.10	0.1		
Alcohol (units per day)	−0.01	0.9		
Medication use (yes vs. no)				
Antibiotics	−0.12	0.1		
Immunosuppressive medication (yes vs. no)				
Prednisolone	−0.40	<0.001		
Mycophenolate mofetil	−0.36	<0.001	−0.18	0.014
Tacrolimus	−0.27	<0.001		
Cyclosporine	−0.06	0.4		
Azathioprine	0.00	1.0	−0.10	0.1
Everolimus	0.04	0.5		
Statins	−0.15	0.024		
Proton pump inhibitors	−0.26	<0.001		
Macrogol	0.06	0.4		
Lactulose	0.10	0.1		
Loperamide	0.02	0.8		
Biguanide drugs	−0.04	0.6		
Insulin	−0.05	0.5		
Antidepressants	−0.14	0.044		
Primary renal disease of RTR (yes vs. no)				
Unknown	−0.04	0.6		
Inflammatory disease	0.02	0.8		
Congenital and hereditary kidney disease	0.00	1.0		
Renal vascular disease, excluding vasculitis	0.09	0.3		
Diabetes Mellitus	−0.08	0.3		
Others (yes vs. no)				
Suffering from Diabetes Mellitus	−0.06	0.4		
History of allograft rejection	−0.02	0.8		

Abbreviations: BMI, body mass index; eGFR, estimated glomerular filtration rate; CH_4_, methane; H_2_, hydrogen; *M. smithii*, *Methanobrevibacter smithii*; St. β, standardized beta. *R*^2^ = 0.550. * Run backwards.

## Supplementary Materials

The following are available online at https://www.mdpi.com/2077-0383/9/2/518/s1, Supplementary File 1: extended description of the methods used to quantify the number of M. smithii in fecal samples, Supplementary File 2: data distributions of breath H_2_ and CH_4_ concentrations, Supplementary data: raw data.
