# Supplementary material for: Altered Gut Microbial Fermentation and Colonization with Methanobrevibacter smithii in Renal Transplant Recipients
Source: J Clin Med. 2020 Feb 14;9(2):518. doi: 10.3390/jcm9020518 (PMC7073595; doi:10.3390/jcm9020518)
Supplement: Supplementary file 1 [file jcm-09-00518-s001.zip › New jcm-690135 supplementary/Supplementary_File_1_jcm-690135.docx]

Supplementary File 1

Extended description of the methods used to quantify the number of M. smithii in fecal samples.

3.1.1.DNA isolation from faeces.

DNA from faecal samples was isolated using the RBB and Qiagen method, also known as the double bead-beater method, it was adapted from Yu *et al*. with modifications described by de Goffau *et al*. [1,2]. 0.25 g of faecal sample was weighed in a screw-cap tube with 4 glass beads of 4mm and a total of 0.5 gram of 0.1 and 0.5 mm Silica/Zirconia beads (BioSpec), to which 1 ml of Lysis buffer(500mM NaCl, 50 mM Tris-HCL, 0.5 M EDTA, 10% SDS and MilliQ) was added. Samples were treated by bead beating (Precellys 24, Bertan Technologies) at 5.5 ms for 3 times 1 minute, with 30 sec pauses in between. Afterwards the samples were heated at 95°C for 15 minutes and shaken every 5 minutes. The samples were then put on ice for 5 minutes and centrifuged at 4°C for 5 minutes at full speed. The supernatant was transferred into a new Eppendorf tube and 300 µl lysis buffer was added to the screw-cap tubes, after which the bead beating steps were repeated and the supernatants were pooled. The QIAamp DNA stool mini kit from Qiagen was used for the clean-up of the DNA combined with ammonium acetate precipitation of debri. In every sample one InhibitEx tablet from the kit and 260 µl of 10M ammonium acetate was added (Sigma-Aldrich). The samples were vortexed and incubated on ice for 5 minutes. Afterwards the samples were centrifuged at 4°C at full speed for 10 minutes. The supernatant was transferred to a new Eppendorf tube. Again 260 µl of ammonium acetate was added to the supernatant and the previous 2 steps were repeated.

The supernatant that was obtained was transferred to two Eppendorf tubes and the same volume of cold isopropanol was added. This was mixed and incubated on ice for 30 minutes. The samples were then centrifuged for 15 minutes at 4°C at full speed. The supernatant was removed by decanting and most of the moisture was removed by tapping. The pellet was washed with 500µl 70 % cold ethanol EtOH for 2 minutes. Afterwards, the pellet was dried to air with the cups upside down. Each pellet was dissolved in 100 µl of AE buffer from the kit and stored overnight at 4°C. The pellets are pooled together and two µl of DNase free RNase (500u Roche) was added to every sample. Secondly, they were incubated for 15 minutes at 37°C. Then 15 µl of proteinase K was added to every sample and mixed well. Afterwards 200 µl of Buffer AL was added and mixed and the samples were incubated at 70°C for 10 minutes thereafter the samples were then put on ice for 1 minute. Then 200 µl of -80°C 100% ETOH were added and mixed well. The samples were transferred to a QIAmp column and centrifuged for 1 minute at 4°C at full speed. The column was put in a new collection tube and 500 µl of Buffer AW1 was added. Again, the samples were centrifuged for 1 minute at 4°C at full speed. After this, the column was put in a new collection tube and 500 µl of Buffer AW2 was added and centrifuged for 1 minute at 4°C at full speed. The column was dried by centrifugation for 1 minute at 4°C and further dried by air for 1 minute Next 100 µl of Buffer AE was added to the columns and incubated for 1 minute. Afterwards the samples were centrifuged at full speed for 1 minute at 4°C. The elute was re-used by decanting back in the column and incubated for 1 minute and then centrifuged at full speed for 1 minute. The elute was put in an Eppendorf tube and the concentration of the DNA was measured with the Nanodrop-ND2000 (Thermo Scientific). The DNA was stored at -25°C [3].

3.1.1. Real-time quantitative PCR assay development

To find the range of quantification, first the standard curve was made. A set of primers was designed to detect the nif*H* gene of the M. smithii in the total DNA of the sample. A second set of primers and a probe taken from a published article by Johnston et al. were used to target a 151 segment of the nif*H* gene [4]. Both sets of primers are described in Table A1. This second set of primers was applied to the sample with a normal PCR and from these the PCR results a standard curve was diluted to form a standard curve with log dilutions ranging from 10-1000000 ag.

Table S1: Primer and probe sequences.

| **Oligonucleotide** | **Sequence** | **Location*** |
| --- | --- | --- |
| NifH forward† | 5’-CAC GTA CAT TGT GCG GTA GA-3’ | 64-83 |
| NifH Reverse† | 5’-TCC ATA GGT TCC GGT GTT GTG-3’ | 685-665 |
| Mnif 202F‡ | 5’-GAA AGC GGA GGT CCT GAA-3’ | 202-219 |
| Mnif 353R‡ | 5’-ACT GAA AAA CCT CCG CAA AC-3’ | 334-353 |
| Mnif Probe‡ | 5’-[FAM]- CCG GAC GTG GTG TAA CAG TAG CTA-[BHQ-1]-3’ | 236-259 |

BHQ-1=black hole quencher 1. * The nucleotide position in reference to the *M. smithii* gene sequence (accession no. CP000678, region 1752235 … 1753032). † Sequences that are designed in this study. ‡ Sequences taken from Johnston *et al* [4].

| 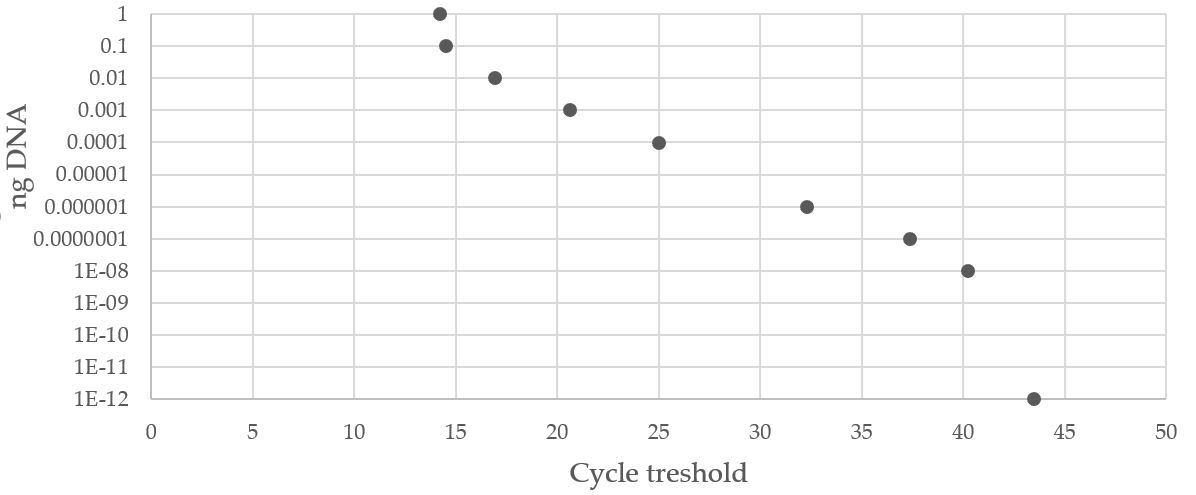 |
| --- |

**Figure S1.** Standard curve. Relationship between the initial DNA and the Cycle treshold values from each standard. In the graph that can be made using our results, the linear part is the range in which samples can be quantified. The linear part ranging from a cycle threshold value of 17 to 40, with a R^2^ of 0.99.

The thermal cycling and fluorescence detection were carried out in the 7500 Fast real-time PCR system (Applied Biosystems). The reaction was performed with a total volume of 25 µl containing 12.5 µl of the 2x universal mastermix (containing ROX reference dye ,Taqman), 0.5 µl of BSA was added with 8.5 µl of RNA und DNA free water,300 nM of both the Nif*H* and Mnif primers, 100 nM probe and between 13 to 300 ng of DNA from the faecal sample or 3 µl of the quantification standard. All samples were run in threefold with a 7 point standard curve which served as a positive control, with a blanc also in threefold. The final cycling conditions were an initial step of 95º Celsius for 10 minute, and with a 50 times repeated cycle starting with 10 seconds at 95º degrees and then for 30 seconds at 57º degrees Celsius. The data from the RT-PCR was processed with the use of the program SDSShell (Applied Biosystems). This is linked with the Taqman machine that has been used (7500 fast real time PCR system, Applied Biosystems).The baseline and threshold were set automatically by the SDSShell software. The Cycle threshold (Ct) where the fluorescence of the sample exceeded the background fluorescence were recorded. The numbers of M. smithii were calculated from the standard curve. This standard curve was produced by the quantification standard in relation to their Ct value.

**References**

1. Yu, Z.; Morrison, M. Improved extraction of PCR-quality community DNA from digesta and fecal samples. *Biotechniques* **2004**, *36*, 808–812.

2. Goffau, M.C. De; Luopajärvi, K.; Knip, M.; Ilonen, J.; Ruohtula, T.; Härkönen, T.; Orivuori, L.; Hakala, S.; Welling, G.W.; Harmsen, H.J. Fecal Microbiota Composition Differs Between Children With b -Cell Autoimmunity and Those Without. *Diabetes* **2013**, *62*, 1238–1244.

3. Khelaifia, S.; Garibal, M.; Robert, C.; Raoult, D.; Drancourt, M. Draft Genome Sequencing of Methanobrevibacter oralis Strain JMR01, Isolated from the Human Intestinal Microbiota. *Genome Announc.* **2014**, *2*, 2013–2014.

4. Johnston, C.; Ufnar, J.A.; Griffith, J.F.; Gooch, J.A.; Stewart, J.R. A real-time qPCR assay for the detection of the nifH gene of Methanobrevibacter smithii, a potential indicator of sewage pollution. *J. Appl. Microbiol.* **2010**, *109*, 1946–1956.
