# Supplementary material for: Altered Gut Microbial Fermentation and Colonization with Methanobrevibacter smithii in Renal Transplant Recipients
Source: J Clin Med. 2020 Feb 14;9(2):518. doi: 10.3390/jcm9020518 (PMC7073595; doi:10.3390/jcm9020518)
Supplement: Supplementary file 1 [file jcm-09-00518-s001.zip › New jcm-690135 supplementary/Supplementary_File_2_jcm-690135.docx]

Supplementary File 2

| 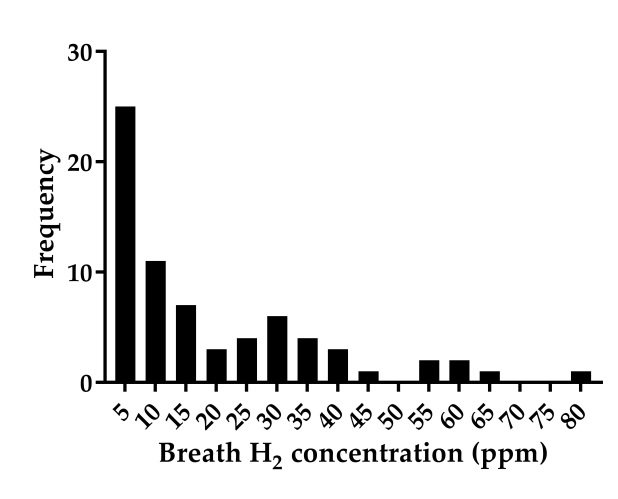  (**a**) | 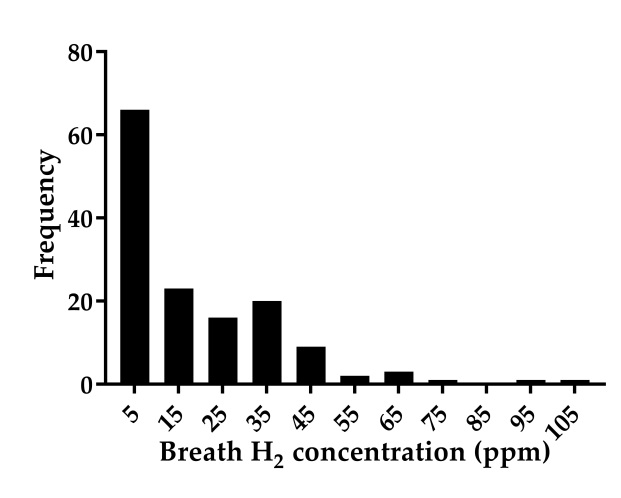  (**b**) |
| --- | --- |

**Figure S2 and S3.** Data distribution of: (**a**) breath H_2_ concentration of HC and (**b**) breath H_2_concentration of RTR. Abbreviations: H_2_, hydrogen; HC, healthy controls; RTR, renal transplant recipients.

| 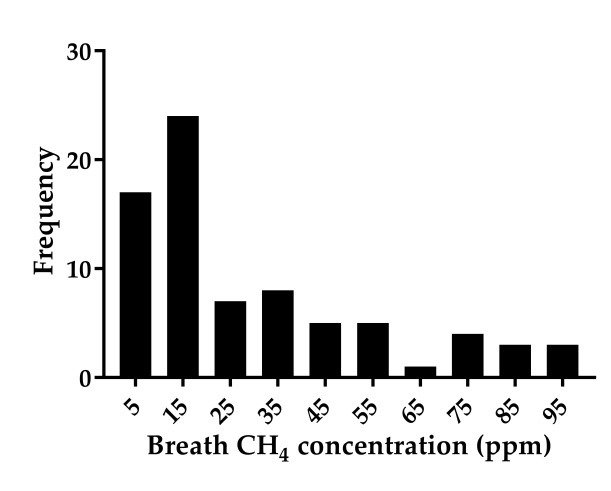  (**a**) | 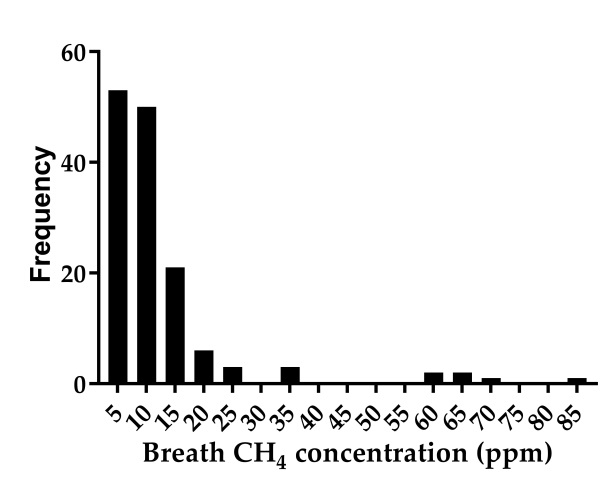  (**b**) |
| --- | --- |

**Figure S4 and S5.** Data distribution of: (**a**) breath CH_4_ concentration of HC and (**b**) breath CH_4_ concentration of RTR. Abbreviations: CH_4_, methane; HC, healthy controls; RTR, renal transplant recipients.
